# Inhibition of apoptotic Bax translocation to the mitochondria is a central function of parkin

**DOI:** 10.1038/cddis.2014.278

**Published:** 2014-07-03

**Authors:** R A Charan, B N Johnson, S Zaganelli, J D Nardozzi, M J LaVoie

**Affiliations:** 1 Harvard Medical School, Boston, MA, USA; 2 Department of Neurology, Center for Neurologic Diseases, Brigham and Women's Hospital, Harvard Medical School, Boston, MA, USA

## Abstract

Parkinson's disease (PD) is the second most prevalent neurodegenerative disorder, affecting 1–3% of the population over 65. Mutations in the ubiquitin E3 ligase parkin are the most common cause of autosomal recessive PD. The parkin protein possesses potent cell-protective properties and has been mechanistically linked to both the regulation of apoptosis and the turnover of damaged mitochondria. Here, we explored these two functions of parkin and the relative scale of these processes in various cell types. While biochemical analyses and subcellular fractionation were sufficient to observe robust parkin-dependent mitophagy in immortalized cells, higher resolution techniques appear to be required for primary culture systems. These approaches, however, did affirm a critical role for parkin in the regulation of apoptosis in primary cultured neurons and all other cells studied. Our prior work demonstrated that parkin-dependent ubiquitination of endogenous Bax inhibits its mitochondrial translocation and can account for the anti-apoptotic effects of parkin. Having found a central role for parkin in the regulation of apoptosis, we further investigated the parkin-Bax interaction. We observed that the BH3 domain of Bax is critical for its recognition by parkin, and identified two lysines that are crucial for parkin-dependent regulation of Bax translocation. Last, a disease-linked mutation in parkin failed to influence Bax translocation to mitochondria after apoptotic stress. Taken together, our data suggest that regulation of apoptosis by the inhibition of Bax translocation is a prevalent physiological function of parkin regardless of the kind of cell stress, preventing overt cell death and supporting cell viability during mitochondrial injury and repair.

Loss-of-function mutations in the ubiquitin E3 ligase parkin are the most common cause of autosomal recessive Parkinson's disease (PD).^1^ Multiple functions have been ascribed to parkin, most notably the inhibition of apoptosis^2, 3, 4, 5, 6, 7^ and the induction of autophagic mitochondrial turnover (mitophagy).^8, 9^ However, the relative scale of these effects mediated by endogenous parkin and whether these processes can occur concomitantly or are mutually exclusive, is not known.

Bax is a primary effector of cell death that translocates from the cytosol to the mitochondria upon stress, where it facilitates cytochrome *c* release and the subsequent caspase cascade.^10^ We previously identified Bax as a parkin substrate, and found that the anti-apoptotic effects of parkin can be directly linked to the parkin-dependent ubiquitination of Bax and inhibition of its mitochondrial translocation.^3^ Recent corroborative evidence showed that primary cultured neurons from parkin knock-out (KO) mice accumulate greater levels of activated Bax at the mitochondria than wild-type (WT) neurons after apoptotic stimulation,^11^ while a separate report showed the parkin-dependent ubiquitination of Bax during mitophagy.^12^

In addition to its anti-apoptotic function, parkin facilitates a depolarization-induced and autophagy-dependent turnover of mitochondria. This process is robustly observed in immortalized cell lines expressing human parkin, where exposure to the mitochondrial depolarizing agent carbonyl cyanide 3-chlorophenylhydrazone (CCCP) causes rapid recruitment of parkin from the cytosol to the mitochondrial outer membrane and a coordinated proteasome and autophagosome-mediated turnover of the entire organelle.^8, 13, 14, 15^ Examination of this process in primary neuronal cultures with endogenous parkin expression, however, has been challenging,^16, 17, 18, 19^ and a cooperative role for inhibition of mitochondria-dependent cell death has not been investigated in the context of mitophagy.

In this study, we sought further insight into the biological functions of parkin across multiple cell types. Our data showed that whole-cell biochemical techniques were not sufficient to observe the participation of endogenous parkin in mitochondrial turnover but were able to confirm the parkin-dependent regulation of apoptosis. Further examination of the parkin-dependent regulation of apoptosis identified two specific lysines of Bax that are critical for recognition and inhibition of its translocation to the mitochondria by parkin. In addition, the BH3 domain of Bax was critical for its association with parkin. Importantly, we observed parkin-dependent mitophagy and inhibition of apoptotic Bax translocation in the same cell culture systems, suggesting that these two pathways coexist and likely cooperate within neurons. Taken together, our data indicate that the parkin-dependent regulation of Bax is critical for cell survival, irrespective of the nature of cell stress involved.

## Results

### Roles of endogenous parkin in brain-derived primary cells

**Figure 1:**
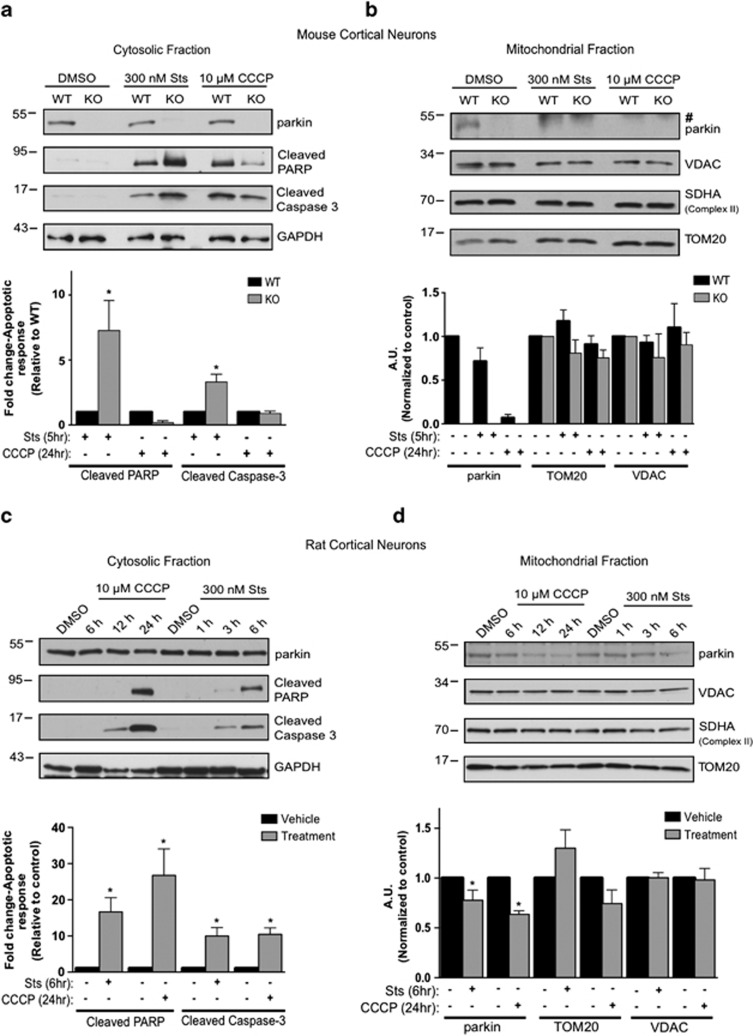
Primary brain-derived cells demonstrate a muted mitophagic response, but show a parkin-dependent sensitivity to apoptosis. Mouse or rat primary cortical neurons isolated at E18 were treated with 300 nM staurosporine or 10 *μ*M carbonylcyanide-3-chlorophenylhydrazone (CCCP) for stated times. (**a**) Cytosolic fractions from WT or parkin KO mouse cortical neurons were treated with DMSO, staurosporine (Sts) for 6 h or CCCP for 24 h and probed for parkin and apoptotic markers, and (**b**) the mitochondrial fractions were analyzed for mitochondrial protein levels. Hash (#) indicates a non-specific band. For quantification, band intensities of proteins in the cytosolic fractions were normalized to WT, and the mitochondrial proteins were normalized to the control (DMSO). (**c**) WT rat cortical neurons were treated with DMSO, CCCP or staurosporine for times listed and probed for parkin, apoptotic markers and (**d**) mitochondrial proteins. For quantification, band intensities of proteins from cytosolic and mitochondrial fractions were normalized to vehicle (DMSO). All bars represent mean±S.E.M., *n*=3. Asterisks indicate significant differences compared with WT or control (*P*<0.05)

**Figure 2:**
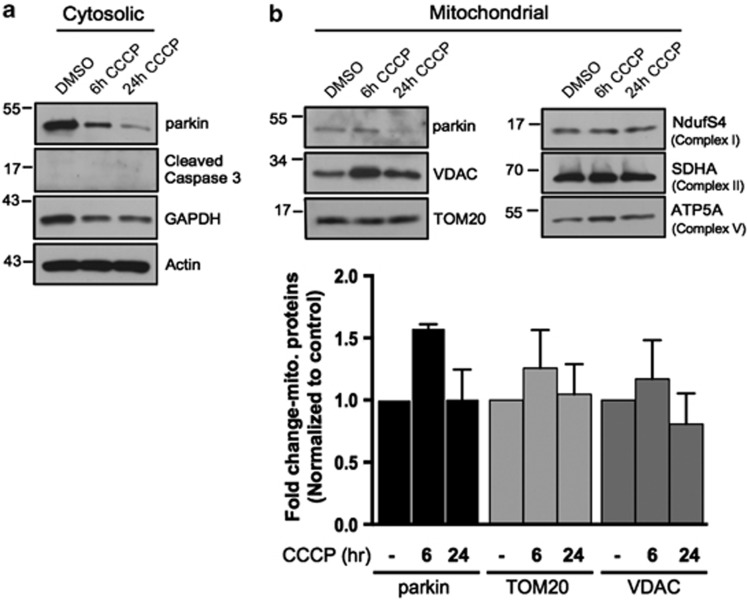
Subcellular fractionation does not reveal a robust mitophagic response to CCCP in primary cultured murine astrocytes. (**a**) WT mouse astrocytes isolated from P1–P4 mice were treated with either DMSO or 20 *μ*M CCCP for 6 or 24 h and lysates from cytosolic and mitochondrial fractions were probed for parkin and apoptotic markers, and mitochondrial protein levels. (**b**) For quantification, band intensities of proteins from mitochondrial fractions were normalized to vehicle (DMSO). All bars represent mean±S.D., *n*=3

We and others have shown that endogenous parkin has an important role in the neuronal regulation of apoptosis.^3, 11, 20^ However, we had not examined the response of endogenous parkin in these cells to induction of mitophagy. Primary cortical neurons from E18 WT and parkin KO mice were treated with vehicle, CCCP (10 *μ*M, 24 h), or staurosporine (300 nM, 5 h). Isolation of cytosolic and mitochondrial fractions revealed that neither treatment induced robust mitochondrial translocation of parkin (Figures 1a and b). Outer mitochondrial membrane proteins voltage-dependent anion channel (VDAC) and translocase of outer membrane 20 (TOM20) were unaffected, indicating these biochemical measures were insufficient to resolve CCCP-induced mitophagy (Figure 1b). Staurosporine treatment increased the apoptotic markers cleaved caspase-3 and cleaved poly ADP ribose polymerase (PARP) in cytosolic fractions of both cultures, however, they were substantially elevated in parkin KO neurons compared with WT (Figure 1a), as expected.^2, 3^ Interestingly, 24-h exposure to CCCP also resulted in evidence of apoptosis in both WT and parkin KO neurons, and while there were subtle differences between WT and KO neurons, these were not statistically significant. To consider species-specific differences, we also examined WT rat primary cortical neurons. Apoptotic stress (300 nM, 5 h) or CCCP (10 *μ*M, 24 h) did not induce quantifiable mitochondrial translocation of parkin and showed a trend for decreased TOM20 that was not statistically significant, while both treatments also increased apoptotic markers (Figures 1c and d). To study an additional brain resident cell that expresses endogenous parkin, we isolated primary astrocytes from WT neonatal mice and assessed the behavior of parkin during mitochondrial stress. We failed to detect mitochondrial translocation of parkin or the turnover of mitochondrial proteins following 6 or 24 h of CCCP treatment (Figures 2a and b). We did observe a time-dependent decrease in parkin levels in the cytosolic fractions, possibly due to stress-induced turnover, decreased parkin solubility or cell death (Figure 2a).

### Scale of CCCP-induced parkin-mediated mitophagy varies by cell type

**Figure 3:**
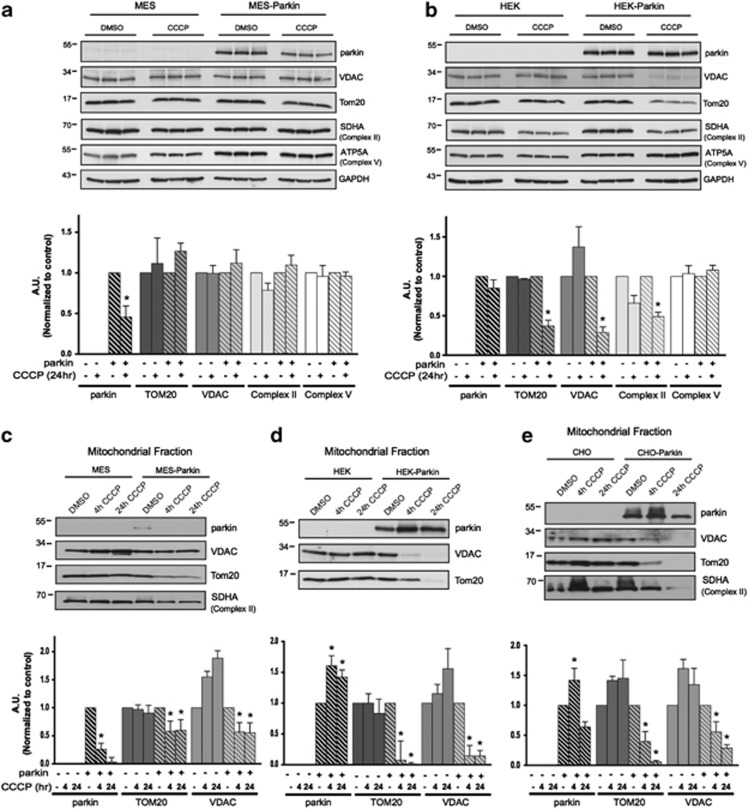
The scale of parkin-mediated mitophagy is affected by cell type. Dopaminergic MES cells (**a**), and HEK cells (**b**), each with or without stable expression of parkin, were treated with either DMSO or 20 *μ*M CCCP for 24 h in triplicate. Whole-cell lysates were probed for outer (VDAC and TOM20) and inner (Complex II and V) mitochondrial proteins to assess mitophagy. For quantification, band intensities of proteins were normalized to vehicle (DMSO). (**c**) MES, (**d**) HEK and (**e**) CHO cells, with or without stable expression of parkin, were treated with either DMSO or 20 *μ*M CCCP for 4 or 24 h, and isolated mitochondrial fractions were probed for parkin translocation and for outer and inner mitochondrial proteins to assess mitophagy. For quantification, band intensities of proteins from mitochondrial fractions were normalized to vehicle (DMSO). All bars represent mean±S.D., *n*=3. Asterisks indicate significant differences compared with control (*P*<0.05)

Our inability to resolve mitophagy using biochemical techniques in neurons and astrocytes led us to speculate that the proportion of the mitochondrial network turned over during CCCP-induced mitophagy might differ across cell types. We first assessed MES neural cell lines, originally derived from the dopaminergic neurons of the rat substantia nigra displaying dopaminergic properties and expressing low levels of endogenous parkin.^21, 22^ MES cells with or without stable overexpression of WT human parkin were treated with vehicle or 20 *μ*M CCCP for 24 h. In whole-cell lysates we did not observe robust CCCP-induced changes in the levels of outer mitochondrial proteins (VDAC, TOM20) or the inner mitochondrial membrane proteins (SHDA (member of Complex II) and ATP5A (member of Complex V)), regardless of parkin expression (Figure 3a). However, the same treatment of human embryonic kidney 293 (HEK) cells which also express low endogenous parkin^23^ with stable parkin expression (HEK-Parkin) revealed a significant reduction in several (but not all) markers of mitochondrial content in the whole-cell lysates of HEK-Parkin, but not HEK cells (Figure 3b), demonstrating a more pronounced mitophagy in this cell type. It is unclear why ATP5A was not reduced in HEK-Parkin cells, as premature time points, selective mitochondrial protein degradation^24, 25^ or other mechanisms may be responsible.

Next, we analyzed mitochondrial fractions of MES, HEK and Chinese hamster ovary (CHO) cells with or without stable parkin overexpression at 4 or 24 h. In MES-Parkin cells, we did not observe mitochondrial translocation of parkin at either time points (Figure 3c). In fact, mitochondrial levels of parkin were reduced after CCCP treatment, consistent with observations in the primary neuronal and astrocyte cultures (Figures 1d and 2a). However, when analyzing isolated mitochondrial fractions, a subtle and significant decrease in TOM20 and VDAC was observed in MES-Parkin cells (Figure 3c) that was not resolved at the whole-cell level (Figure 3a), indicative of a mitophagic response in these neural cells comparable to the subtle loss of TOM20 observed in rat primary cortical neurons. Mitochondrial fractions from HEK-Parkin and CHO-Parkin cells, however, displayed a more robust response. We observed translocation of parkin to the mitochondria after 4 h of CCCP treatment (Figures 3d and e), and reduction in TOM20 and VDAC as early as 4 h post treatment. In fact, these markers were virtually absent at 24 h (Figures 3d and e), suggesting complete mitochondrial turnover in these cells.^26^

### Regulations of Bax and mitochondrial turnover are not mutually exclusive functions of parkin

**Figure 4:**
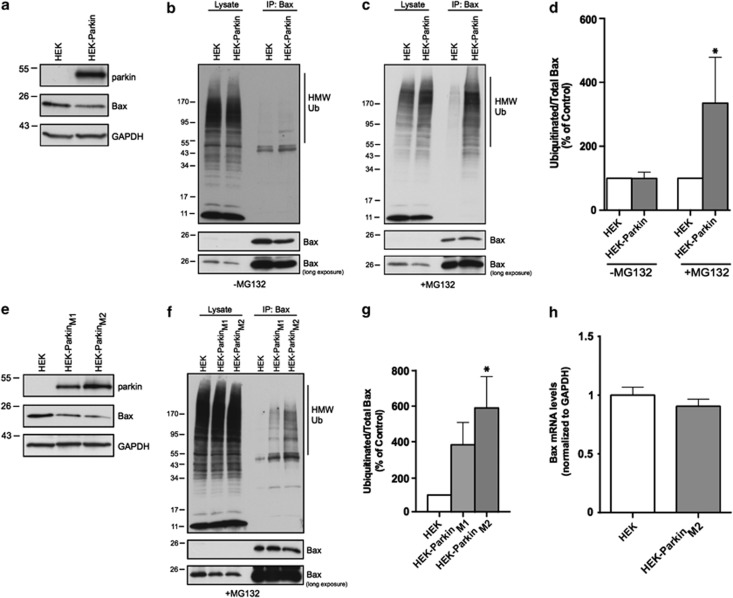
Parkin-mediated regulation of Bax is cell-type independent. (**a**) Endogenous Bax expression in whole-cell lysates of HEK cells in a polyclonal line with stable expression of parkin (HEK-Parkin). HEK and HEK-Parkin cells either untreated (**b**) or treated with proteasome inhibitor (MG-132, 10 *μ*M) for 6 h (**c**). Following lysis, whole-cell lysates and Bax IPs were probed by Western blot for ubiquitin. (**d**) Quantification of IPed ubiquitin levels from (**b**) and (**c**) plotted as a percentage of control (HEK). (**e**) Endogenous Bax expression in lysates from two monoclonal lines (M1 and M2) expressing differential levels of parkin. (**f**) HEK, HEK-Parkin_M1_ and HEK-Parkin_M2_ cells were treated with proteasome inhibitor (MG-132, 10 *μ*M) for 6 h. Cells were lysed, and whole-cell lysates and Bax IPs were probed by Western blot for ubiquitin. (**g**) Quantification of IPed ubiquitin levels from **b** and **c** plotted as a percentage of control (HEK). (**h**) Quantitative RT-PCR was performed in HEK and HEK-Parkin_M2_ cells to assess Bax mRNA levels. Graph is plotted as Bax mRNA levels normalized to control GAPDH mRNA levels. All bars represent mean±S.D., *n*=3. Asterisks indicate significant differences compared with control (*P*<0.05)

We previously described the ubiquitination and regulation of endogenous Bax by parkin in neural MES cells and primary cultured neurons.^3^ Given that the scale of mitophagy appeared different across various cell types, we considered whether mitophagy is preferentially manifested in some cell types and the inhibition of Bax translocation in others. In the HEK-Parkin cell line that displayed the most robust mitophagy, the levels of Bax in whole-cell lysates were also dramatically reduced by parkin (Figure 4a), suggesting that the participation of one parkin function does not preclude the other. Next, we treated HEK and HEK-Parkin cells with vehicle or the proteasomal inhibitor MG-132 (10 *μ*M, 6 h), and probed for levels of ubiquitinated Bax by immunoprecipitation (IP)–Western blot. In the absence of MG-132 we did not detect ubiquitinated Bax (Figures 4b and d), however, MG-132 treatment allowed for detection of endogenous ubiquitinated Bax, which was substantially increased by parkin expression (Figures 4c and d). We then isolated two monoclonal parkin-expressing lines from the polyclonal pool with differential parkin expression, named clones M1 and M2 (Figure 4e). We not only confirmed a gene-dose response for the effects of parkin on whole-cell Bax levels (Figure 4e) but also for the levels of ubiquitinated Bax in these respective lines (Figures 4f and g).

Prior studies have suggested that parkin may have a role in gene transcription.^27, 28, 29^ Given the large effect on endogenous Bax by parkin, we measured the mRNA levels of Bax by qPCR, using the HEK-Parkin_M2_ clone that expressed the highest levels of parkin and had the greatest effect on Bax protein levels. Despite the dramatic changes in Bax protein, we found no change in Bax mRNA levels when normalized to GAPDH (Figure 4h).

### The BH3 domain of Bax is required for its interaction with parkin

**Figure 5:**
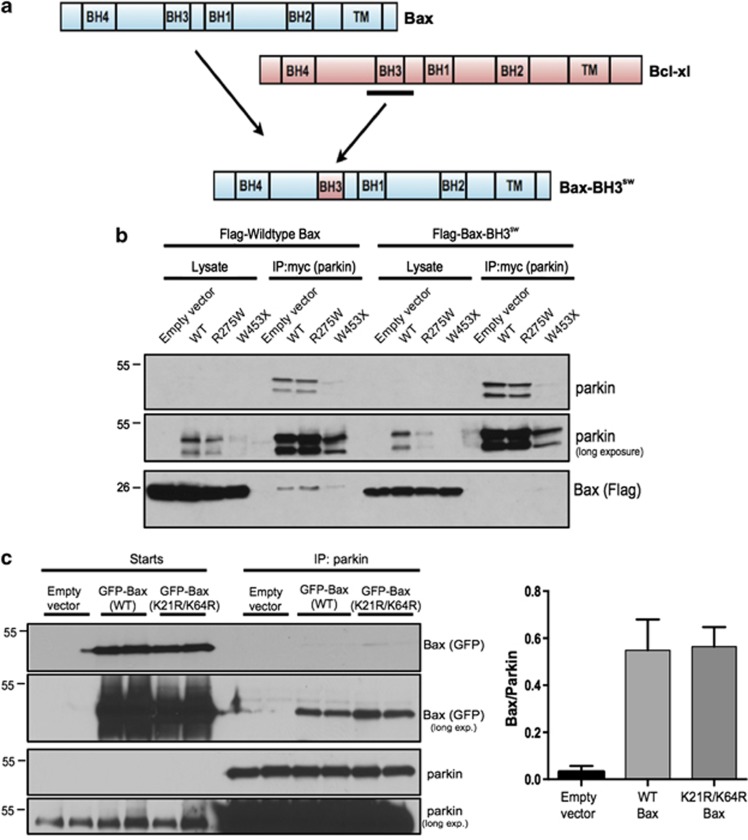
Swapped Bax BH3 domain causes reduction in the parkin-Bax interaction. (**a**) Schematic of Bax and Bcl-xl structures and the engineered construct with the BH3 domain swapped with the BH3 domain of Bcl-xl (Bax-BH3^sw^). (**b**) Flag-tagged wild-type Bax or Bax-BH3^sw^ were transiently co-transfected in HEK cells along with empty vector, myc-tagged wild-type parkin (WT) or mutant parkin (R275W and W453X). Whole-cell lysates were subjected to IP of parkin using a myc-tag antibody and whole-cell lysates and IPs were probed for Bax using a Flag-tag antibody to visualize co-immunoprecipitation along with parkin to demonstrate pulldown. (**c**) GFP-tagged WT or K21R/K64R Bax was transiently transfected in CHO cells stably expressing parkin (CHO-Parkin). Whole-cell lysates were subjected to IP using a parkin antibody, and starting lysates and IPs were probed for Bax using a GFP antibody to visualize co-immunoprecipitation along with parkin to demonstrate pulldown. For quantification, band intensities of WT and mutant Bax were normalized to levels of parkin pulldown. All bars represent mean±S.D., *n*=5

Having confirmed the potent effect of parkin on Bax at the protein level, we sought new insights into the nature of their physical interaction. Mutagenesis and truncation/deletions of parkin often lead to its misfolding and insolubility,^30^ hence we focused on subtle modifications of Bax. The BH3 domain of Bax is required for its pro-apoptotic properties,^31^ and other putative substrates of parkin (Nix, Bcl-2) also contain this motif.^32, 33^ Thus, we hypothesized that this domain is important for parkin–Bax interactions. Since Bcl-xl does not interact with parkin in mammalian cells,^32^ we swapped the full α-helix 2 of Bax containing the BH3 domain with the complete α-helix 2 of the anti-apoptotic Bcl-xl containing its BH3 domain (Bax-BH3^sw^) in an effort to preserve proper folding (Figure 5a). We then assessed if this replacement affected parkin binding. Catalytically inactive E3 ligase mutants are known to bind their substrates more avidly than their active forms,^3, 34^ thus, we analyzed WT parkin along with a PD-linked missense mutation (R275W) and a premature termination (W453X). HEK cells were co-transfected to express one of the parkin variants along with either Flag-tagged WT Bax or Bax-BH3^sw^ and the two proteins were co-IPed. We observed comparable levels of parkin expression and pulldown across all conditions for WT and R275W mutant, while the W453X mutation was not well expressed or IPed (Figure 5b), as described earlier.^35, 36^ We observed that WT Bax interacted with WT parkin, and that the PD-linked loss-of-function mutant R275W pulled down more WT Bax despite a comparable parkin IP (Figure 5b), as previously described.^3^ However, the interaction between parkin and Bax was lost in all cases when the BH3 domain of Bax was replaced (Figure 5b).

### Two lysines are required for inhibition of mitochondrial Bax translocation by parkin

Prior work demonstrated that the parkin-dependent regulation of Bax was dependent on the availability of lysines within Bax.^3^ The BH3 domain of Bax is important for parkin recognition (Figure 5), and its secondary structure reveals that the sole lysine in this domain (K64) is solvent exposed.^37^ Additionally, a recent study identified K21 on the N terminus of Bax to be ubiquitinated by parkin upon CCCP-induced mitophagy.^12^ Hence, we tested both K21 and K64 of Bax for their role in parkin-dependent regulation of mitochondrial translocation. Simultaneous mutation of all nine lysines residues of Bax does not alter its apoptotic function,^3^ thus we mutated K21 and/or K64 to arginines (K21R, K64R and K21R/K64R), preserving charge and likely retaining Bax function. In addition, co-IP analysis revealed that these lysine mutations had no effect on the physical interaction with parkin (Figure 5c).

**Figure 6:**
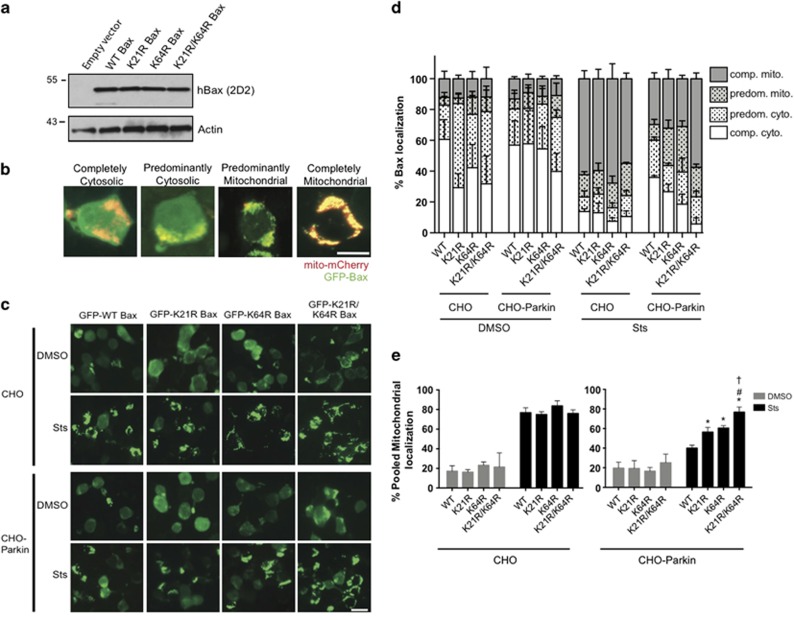
Lysine 21 and 64 of Bax are required for parkin regulation of Bax translocation into the mitochondria. (**a**) Whole-cell lysates of CHO cells transiently expressing wild-type human Bax (hBax) or each of its mutants and probed with the human-specific 2D2 antibody by Western blot. (**b**) Representative images of cells co-transfected with mito-mCherry (mitochondrial marker) and GFP-Bax, showing the various stages of Bax translocation into the mitochondria after treatment with 1 *μ*M staurosporine for 5 h. Scale bar, 10 *μ*m. (**c**) CHO cells without or with stable expression of parkin (CHO-Parkin) were transiently transfected with wild-type GFP-Bax or each of its GFP-tagged mutants. Representative images of cells showing Bax translocation into the mitochondria after treatment with DMSO or 1 *μ*M staurosporine (Sts) for 5 h. Scale bar, 20 *μ*m. (**d**) Quantification of **b**. Cells were scored visually for Bax translocation into the mitochondria as completely cytoplasmic (open), predominantly cytoplasmic (open dotted), predominantly mitochondrial (gray) or completely mitochondrial (gray dotted) by a blinded observer. Hundred and five to 160 cells were counted per condition. Data were plotted from three independent experiments. (**e**) Percentage of pooled mitochondrial fractions (predominantly mitochondrial+completely mitochondrial) plotted from **d**. All bars represent mean±S.D., *n*=3. Asterisk indicates significant differences when compared with WT, # indicates significant difference between K21R and K21R/K64R, † indicates significant difference between K64R and K21R/K64R (*P*<0.05)

Bax is known to translocate to the mitochondria to facilitate apoptosis^38, 39^ and we have observed this response, as well as the parkin-dependent inhibition of Bax translocation, in CHO cells.^3^ Therefore, we transiently transfected CHO cells with GFP-tagged WT Bax or the mutants (K21R, K64R, K21R/K64R), analyzed the lysates and confirmed that the engineered mutations did not affect Bax expression (Figure 6a). The mitochondrial translocation of the expressed Bax constructs was assessed by co-transfecting mito-mCherry to label mitochondria prior to image analysis, as previously described.^3^ Following treatment with vehicle or staurosporine (1 *μ*M, 5 h) to induce apoptosis, we classified GFP-Bax localization as either (i) completely cytosolic, (ii) predominantly cytosolic, (iii) predominantly mitochondrial or (iv) completely mitochondrial (Figure 6b). In parental CHO cells, staurosporine induced rapid translocation of all Bax variants (Figure 6c) with the majority of cells displaying complete mitochondrial translocation (Figure 6d). In CHO-Parkin cells, however, only WT Bax was retained in the cytosol following the induction of apoptosis. Parkin expression was associated with modest reductions in both the K21R and K64R Bax translocation. The K21/K64 double mutant, however, appeared completely unaffected by parkin (Figures 6c and d). For quantitative assessment and statistical analyses, we chose to pool the ‘predominant' and ‘complete' mitochondrial values to encompass all apoptotic Bax translocation. This grouping revealed that the lysine mutations had no effect on Bax localization in the absence of stress, and this was true in both the CHO and CHO-Parkin cell lines (Figure 6e). We also confirmed the potent parkin-dependent inhibition of WT Bax translocation, and the inability of parkin to prevent the translocation of the K21R/K64R Bax to the mitochondria (Figure 6e). Moreover, there was a significant increase in the mitochondrial translocation of the K21R and the K64R mutant as compared with WT Bax, suggesting a partial loss of parkin recognition with each single mutation, whereas the effect of the double mutant appeared to be additive (Figure 6e).

**Figure 7:**
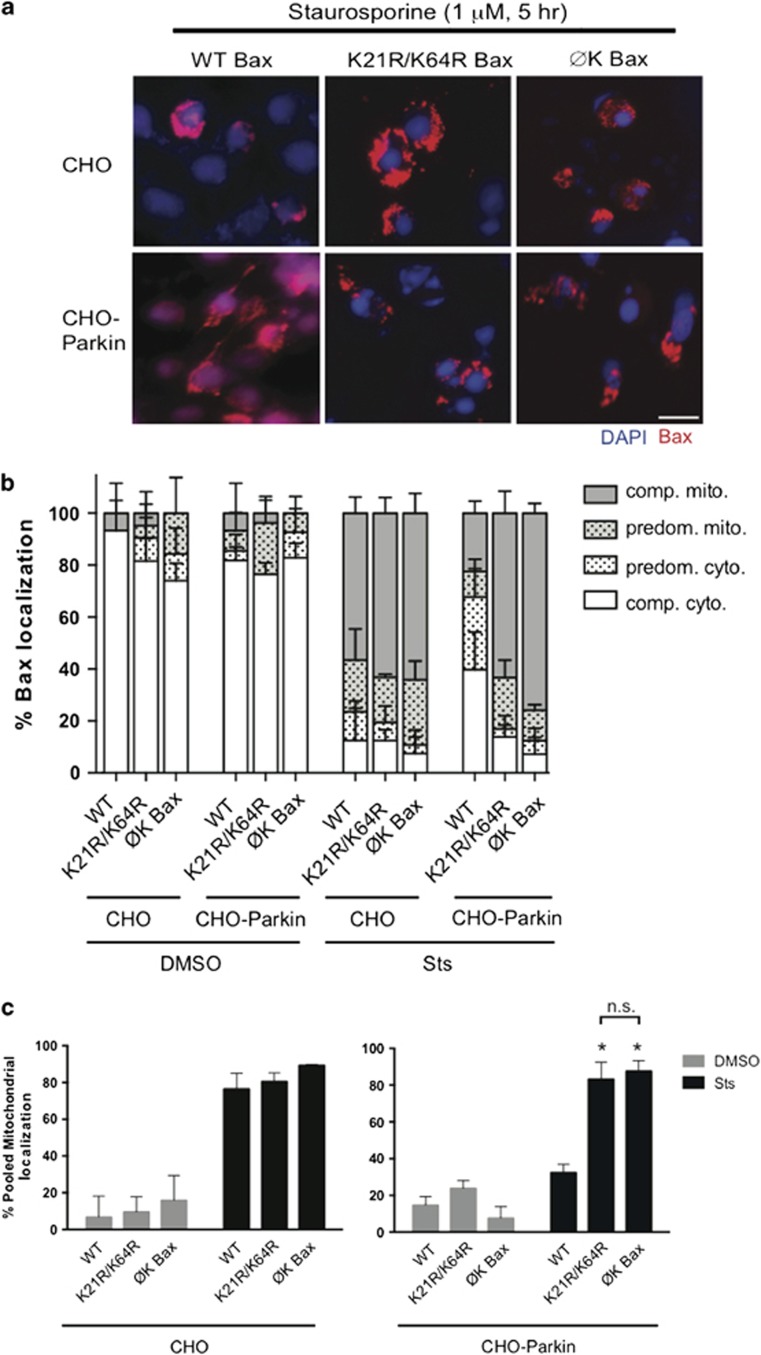
K21 and K64 are necessary and sufficient for parkin-regulated Bax translocation to the mitochondria. (**a**) CHO cells without or with stable expression of parkin (CHO-Parkin) were transiently transfected with untagged wild-type (WT) Bax, Bax with no lysines (ØK Bax) and K21R/K64R Bax. Representative immunocytochemical images of cells showing Bax translocation into the mitochondria (human Bax-specific 2D2 antibody) and after DAPI treatment with 1 *μ*M staurosporine (Sts) for 5 h (**b**) Quantification of **a**. Cells were scored visually for Bax translocation into the mitochondria as completely cytoplasmic (open), predominantly cytoplasmic (open dotted), predominantly mitochondrial (gray) or completely mitochondrial (gray dotted) by a blinded observer. Sixty-six to 102 cells were counted per condition. Data plotted from 3 independent experiments. (**c**) Percentage of pooled mitochondrial fractions (predominantly mitochondrial+completely mitochondrial) plotted from **b**. All bars represent mean±S.D., *n*=3. Asterisks indicate significant differences when compared with WT, n.s. indicates not significant (*P*<0.05)

Having identified two lysines critical for parkin-dependent Bax inhibition, we asked whether these were sufficient to account for the totality of its parkin-dependent regulation. We previously showed that a lysine-null Bax (ØK-Bax) retains its full apoptotic function, but that the anti-apoptotic effect of parkin is completely lost in cells that express this protein.^3^ CHO and CHO-Parkin cells were transiently transfected with untagged WT Bax, ØK Bax or the K21R/K64R Bax mutant and treated with vehicle or staurosporine (1 *μ*M, 5 h). Immunocytochemistry with a human-specific Bax antibody was used to score mitochondrial translocation. Parkin was unable to prevent mitochondrial translocation of the K21R/K64R and the ØK Bax mutant, as opposed to the WT Bax protein, which was retained in the cytosol in the presence of parkin (Figures 7a and b). There no difference between the localization of the double lysine mutant or the lysine-null Bax (Figure 7c), suggesting that K21 and K64 are the sole lysines necessary for parkin-dependent regulation of Bax.

### A pathogenic loss-of-function mutation in parkin fails to inhibit mitochondrial Bax translocation

**Figure 8:**
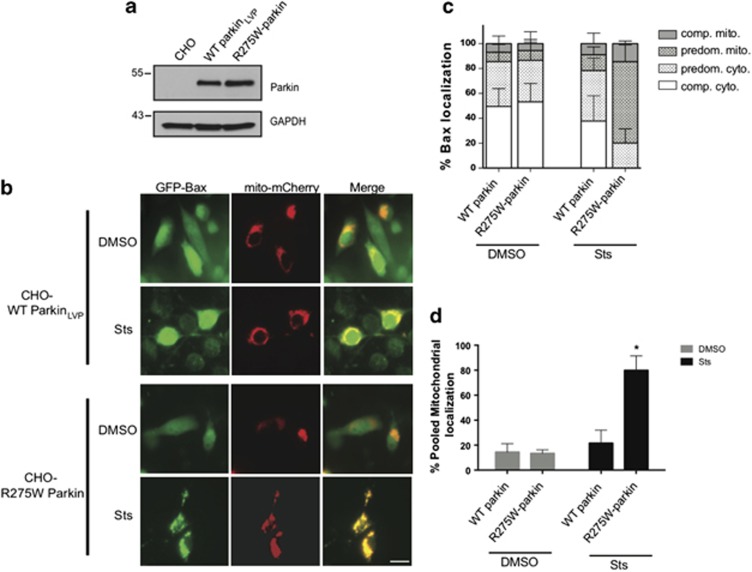
Disease-associated mutation of parkin is unable to regulate Bax translocation to the mitochondria. (**a**) Lysates from CHO cells stably expressing WT parkin or mutant R275W-parkin. (**b**) CHO cells expressing WT or R275W Parkin were transiently transfected with WT GFP-Bax and the mitochondrial marker mito-mCherry. Representative images of fixed cells showing localization of GFP-Bax after treatment with DMSO or 1 *μ*M staurosporine (Sts) for 5 h. Scale bar, 20 *μ*m. (**c**) Quantification of **b**. Cells were scored as described in Figure 5. Forty-six to 64 cells were counted per condition. Data plotted from three independent experiments. (**d**) Percentage of pooled mitochondrial fractions (predominantly mitochondrial+completely mitochondrial) plotted from **b**. All bars represent mean±S.D., *n*=3. Asterisk indicates significant difference when compared with WT (*P*<0.05).

There are numerous disease-linked missense mutations in parkin but many suffer from aggregation, instability and misfolding.^35, 36^ One mutation that has proven amenable for cell biological experimentation is the really interesting new gene 1 (RING1) domain mutant, R275W.^2, 3, 40^ Therefore, WT and R275W-parkin-stable CHO cells (Figure 8a) were transiently co-transfected with GFP-tagged WT Bax and mito-mCherry. Twenty-four hours later, cells were treated with vehicle or staurosporine (1 *μ*M, 5 h), and images were captured post fixation as described above. We observed the expected retention of Bax in the cytoplasm of CHO-Parkin cells following the induction of apoptosis. However, staurosporine induced robust mitochondrial translocation of Bax in the CHO-R275W cells (Figure 8b), despite the comparable expression of this disease-linked mutant. Analysis of endogenous Bax localization revealed that Bax was predominantly at the mitochondria in cells expressing R275W-parkin, whereas it was predominantly cytosolic even after staurosporine treatment in the WT parkin cells (Figure 8c). Statistical analyses of pooled mitochondrial translocation confirmed that WT parkin prevented Bax from accumulating at the mitochondria, whereas the disease-linked parkin mutant did not (Figure 8d).

## Discussion

Here, we describe a varied scale of responses while studying the role of PD-linked ubiquitin E3 ligase parkin in mitochondrial turnover across cell types. Data from multiple primary cultured cells derived from brain suggested a more refined mitophagic response than the near complete loss of mitochondrial material observed in immortalized cell culture systems, but a uniform regulation of Bax by parkin. HEK and CHO cell lines overexpressing parkin showed robust parkin-dependent mitophagy, as well as a potent inhibition of the apoptotic translocation of Bax from the cytosol to the mitochondria. Therefore, these two pathways are not mutually exclusive rather parkin-dependent mitophagy, and the inhibition of Bax-dependent cell death coexist and likely cooperate to maintain cell viability. We show for the first time that parkin-dependent regulation of Bax does not involve effects on transcription, but rather requires the E3 ligase activity of parkin and proteasomal degradation, as a pathogenic loss-of-function mutation could not regulate Bax. We also found that two specific lysines in Bax are required for parkin-mediated inhibition of its mitochondrial translocation.

The BH3 domain of Bax is important for its pro-apoptotic function,^31, 41^ and we report that the domain is necessary for its recognition by parkin. Since other cytosolic parkin substrates (Bcl-2^32^ and Nix^33^) also possess BH3 domains, this may indicate a common recognition motif for parkin. Given these data and the recently solved crystal structure of parkin,^42, 43, 44^ our new findings are likely to prove valuable in future efforts to identify the structural requirement for recognition of cytosolic parkin substrates, and may assist in the identification of new substrates.

Parkin-dependent ubiquitination of Bax is essential for the inhibition of its apoptotic translocation to the mitochondria,^3^ but the specific lysine residues involved were unknown. Mutation of either K21 or K64 of Bax alone resulted in only a modest reduction in parkin function; nonetheless, a combined K21/K64 Bax mutant retains its ability to bind to parkin but not subject to parkin-dependent inhibition. In fact, it behaved identically to a previously characterized Bax construct devoid of lysine resides,^3^ thus, other lysine residues in Bax are likely dispensable for parkin function. The K64 residue of Bax exists within the BH3 domain and is solvent exposed,^45^ while K21 within the N terminus is thought to be inaccessible under non-apoptotic conditions.^39, 46, 47^ Since apoptotic activation of Bax results in a conformational change that exposes the N terminus,^48^ we speculate that under non-apoptotic conditions, parkin only partially retains Bax within the cytoplasm by ubiquitinating the sole accessible K64 residue, consistent with parkin-dependent ubiquitination of Bax in the absence of apoptotic stimulation (Figure 4).^3^ However, stress-induced conformational changes in Bax would result in access to both K64 and K21, allowing parkin to potentially inhibit Bax translocation to the mitochondria and promote cell survival.

There is a potential adaptive advantage to this proposed two-tiered regulation of Bax by parkin. Recent studies suggest that members of the Bcl-2 family may have critical cellular roles to play beyond programmed cell death (for review, see Hardwick and Soane^49^). Bax is thought to play a role in supporting bioenergetics, the mitochondrial network^50^ and in synaptic transmission. and long-term depression.^51^ These processes may require physiological levels of Bax at the mitochondria. In our model, parkin can only partially regulate the mitochondrial localization of Bax under normal conditions, where the N terminus is inaccessible, whereas the apoptotic conformation of Bax would be robustly affected by parkin under more pathologic conditions. Future experiments may distinguish the roles of parkin in modulating physiological and pathological functions of Bax, and determine whether this model also applies to other E3 ligases that regulate Bax.^52^

There is an open debate regarding the role of apoptosis and Bcl-2-related proteins such as Bax in adult-onset neurodegenerative disease, such as PD.^53^ For example, Bax was shown to accumulate in nigral neurons from postmortem samples taken from individuals with PD in one study,^54^ but not in another.^55^ However, Bax-rich inclusions in PD brain have been reported.^56, 57^ Also, MPTP-induced neurotoxicity is associated with mitochondrial Bax accumulation *in vivo*, and Bax KO mice are resistant in this PD model.^58^ Other members of the Bcl-2 family such as Nix, PUMA, Bak and Bim have also been implicated in *in vitro* and *in vivo* models of PD.^57, 59, 60, 61^ Thus, our data may implicate deficits in the parkin-dependent regulation of Bax as a contributing factor to the neuronal loss in PD.

Mitophagy has been well-documented by numerous groups in several immortalized cell lines overexpressing parkin.^8, 62^ However, the nature of this process in neurons, which express endogenous parkin, has been somewhat controversial.^16, 19, 63^ Our data suggest that the scale of mitochondrial turnover in primary cells expressing endogenous parkin may be relatively muted when compared with cancer cell lines overexpressing human parkin. This was evident in our studies where mitophagy was readily observed in HEK cells at the whole-cell level, but only following subcellular fractionation in neural MES cells. Subcellular fractionation, in turn, was insufficient to observe mitophagy in primary neurons. Therefore, higher resolution techniques may be required to examine neuronal mitophagy. Phenotypic differences in parkin mitophagy across different cell lines may be due to the unique bioenergetics of each model system,^19^ differential expression of competing deubiquitinating enzymes, PINK1 expression levels, the time points chosen for our studies or other as yet unidentified cofactors. Nonetheless, we have established the role of cytosolic endogenous parkin in the ubiquitination of endogenous Bax in neurons^3^ and in regulation of apoptosis,^2, 4, 7^ and now we demonstrate the coexistence of both parkin-dependent regulation of Bax and mitochondrial turnover pathways within the same cells. This raises the possibility that the inhibition of Bax-dependent cell death by cytosolic parkin provides the opportunity for mitochondrially recruited parkin to mediate mitophagy and recovery following cell injury. The pro-survival effects of cytosolic parkin during the induction of selective mitophagy by the translocated parkin may be particularly critical for the maintenance of post-mitotic cell types such as neurons, and partially explain the unique neuropathological consequences of parkin deficiency in humans.

## Materials and Methods

### Cell culture, plasmids and transfection methods

All cell culture and transfection methods have been previously described.^2, 3, 21, 22^ Codon optimized WT human Bax and engineered mutant human Bax with the BH3 domain swapped for the BH3 domain of Bcl-xl (Bax-BH3^sw^) were synthesized and subcloned into pcDNA3.3 vector with the addition of a FLAG tag by GeneArt (Life Technologies, Grand Island, NY, USA). Mutant human Bax protein with all lysines replaced with arginines (Bax ∅-Lys) was engineered by GenScript (Piscataway, NJ, USA) and subcloned into pcDNA 3.1(+) vector (Invitrogen, Grand Island, NY, USA).^3^ WT GFP-Bax was a kind gift from Dr Richard Youle (NIH, NINDS, Bethesda, MD, USA). Mutations in WT GFP-Bax at lysine 21, lysine 64 or both lysines 21 and 64 replaced with arginines (K21R, K64R and K21R/K64R, respectively) were engineered using site-directed mutagenesis. Primers for K21R: Forward, 5′-gctctgagcagatcatgaggacaggggcccttttgc-3′ Reverse, 5′-gcaaaagggcccctgtcctcatgatctgctcagagc-3′. Primers for K64R: Forward, 5′-gcgagtgtctcaggcgcatcggggacgaactgg-3′ Reverse, 5′-cgtccccgatgcgcctgagacactcgctcagc-3′. The K21R/K64R double mutant was generated sequentially using the same primers.

### Antibodies and reagents

Antibodies used for Western blotting were parkin (PRK8; sc-32282; Santa Cruz Biotechnology, Santa Cruz, CA, USA), Bax (2772; Cell Signaling, Danvers, MA, USA), VDAC (PA1-954A; Cell Signaling), Actin (ab6276; Abcam, MA, USA), TOM20 (sc-11415; Santa Cruz Biotechnology), Cleaved caspase-3 (9661S; Cell Signaling), Cleaved PARP (9544S; Cell Signaling), GAPDH (sc-365062; Santa Cruz Biotechnology), succinate dehydrogenase complex-subunit A (SDHA) (ab14715; Abcam), Human Bax (2D2; sc-20067; Santa Cruz Biotechnology) NdufS4 (ab55540; Abcam) and ATP5A (ab14748; Abcam). Secondary antibodies and ECL-plus were purchased from GE Healthcare (Piscataway, NJ, USA). Following ECL application, blots were exposed to HyBlot Cl autoradiography film (Denville Scientific, Metuchen, NJ, USA). Staurosporine and CCCP were obtained from Sigma-Aldrich (St. Louis, MO, USA).

### Isolation of primary neurons and astrocytes

The isolation of primary cortical neurons was carried out as previously described.^64^ Following 3 days in culture, neurons were treated with vehicle (DMSO), 300 nM staurosporine for 1, 3 or 6 h, or 10 *μ*M CCCP for 6 or 24 h Astrocytes were isolated using a modified version of the protocol previously described.^65^ Briefly, glia were obtained from P1–P4 pups and plated and cultured *in vitro* for 15–20 days. Once the astrocyte monolayer was confluent, astrocytes were isolated from the culture using mild trypsinization. Astrocytes were freshly plated for experiments and treated with 10 *μ*M CCCP for 6 or 24 h.

### Cytosolic and mitochondrial fractionation

To extract whole cell, cytosolic and mitochondrial fractions, cells or neurons were homogenized and mitochondria pelleted as previously described.^2^ Briefly, treated cells were collected, washed in ice-cold PBS and the following isolation procedure was carried out at 4 **°**C. A fraction of the cells were pelleted and lysed in 1% NP-40 with protein inhibitor cocktail, incubated on ice for 25 min followed by centrifugation at 6000 × *g* for 10 min to obtain soluble whole-cell lysates. For subcellular fractions, the remainder of washed pellets was suspended in 1 ml isolation buffer (200 mM sucrose, 10 mM Tris/MOPS, 1 mM EGTA/Tris) and lysed using 20 strokes of a Potter-Elvehjem homogenizer (Wheaton, Milville, NJ, USA). The homogenate was then drawn with an 18.5 G needle and expelled through a 27.5 G needle eight times. The homogenates were centrifuged at 200 × *g* for 5 min to discard the nuclear pellet and debris. The remaining supernatant was centrifuged at 10 000 × *g* for 10 min to obtain the cytosolic fractions in the supernatant. The pellet containing the heavy mitochondrial fraction was lysed in 1% NP-40 with protein inhibitor cocktail and incubated on ice for 25 min. The NP-40 insoluble material was pelted by centrifugation at 10 000 × *g* for 5 min and was discarded and the soluble extracted mitochondrial proteins were used for analysis. All experiments were performed in triplicate and representative images are shown.

### Western blot

Cells were lysed in equal volumes of buffer containing 1% NP-40 with protease inhibitors on ice for 25 min and then centrifuged at 10 000 × *g* for 10 min. Protein assays were carried out on the supernatants using the Pierce BCA Protein Assay kit (Thermo Scientific, Rockford, IL, USA). SDS-Laemmli buffer was added and samples were heated at 65 °C for 5 min. Equal concentrations of each sample were loaded onto Novex Tris-Glycine (Invitrogen) or Criterion Tris-HCl (Bio-Rad, Hercules, CA, USA) pre-cast gels and transferred onto polyvinylidene fluoride membranes (Millipore, Billerica, MA, USA) for Western blotting.

### Immunoprecipitation

To determine the ubiquitination of Bax, HEK cells were treated with 10 *μ*M MG-132 or vehicle (DMSO) for 6 h prior to lysis. Protein normalized lysates were pre-cleared with Protein-A agarose beads (Roche Diagnostics, Indianapolis, IN, USA) for 2 h at 4 °C and then IPed using a polyclonal Bax antibody conjugated to agarose beads (sc-493AC; Santa Cruz Biotechnology) overnight at 4 °C. The beads were washed 3 × 15 min in 1 × STEN buffer (50 mM Tris, pH 7.6, 150 mM NaCl, 2 mM EDTA, 0.2% NP-40) and SDS-Laemmli buffer was added and samples were heated at 65 °C for 5 min. Given the ability of parkin to decrease Bax,^3^ aliquots of each sample were first analyzed to determine the individual Bax IP efficiency, as parkin expression decreases Bax levels. Then, elutes were normalized to equal Bax pulldown and run alongside starting lysates on subsequent gels to examine Bax ubiquitination (ubiquitin, 3936; Cell Signaling) using standard Western blotting conditions. Blots were stripped in 1 × stripping buffer (62.5 mmol/l Tris, pH 6.8, 2% (wt/vol) SDS, 7.6% *β*ME) at 55 °C for 10 min, washed 3 × 10 min in 0.1% PBS/Tween, and probed for human Bax 2D2 to ensure pulldown.

To determine parkin-Bax interactions, HEK cells were transiently transfected with myc-tagged WT parkin, mutant R275W or W453X parkin along with either Flag-tagged WT Bax or domain-swapped Bax-BH3^sw^. Protein normalized lysates were incubated with myc-conjugated agarose beads (A7470; Sigma-Aldrich) overnight at 4 °C. The beads were washed 3 × 15 min in 1 × STEN buffer and elutes and starting lysates were probed for Bax using Flag M2 antibody (A2220; Sigma-Aldrich). Blots were also probed for parkin to ensure pulldown. To further evaluate parkin-Bax interactions, CHO cells overexpressing WT human parkin (CHO-parkin) were transiently transfected with either GFP-tagged WT Bax or K21R/K64R mutant Bax and lysed for analysis. Protein normalized lysates were IPed using Protein-G agarose beads (Roche Diagnostics) and a monoclonal parkin antibody (PRK8) overnight at 4 °C. Beads were washed and eluted as stated earlier and run alongside starting lysates. Lysates and elutes were probed for Bax using GFP antibody (A11122; Invitrogen). Blots were also probed for parkin (sc-30130; Santa Cruz Biotechnology) to ensure pulldown.

### Quantitative PCR

RNA was purified from parental HEK and stably expressing parkin (HEK-Parkin) cells using the PureLink RNA Mini Kit (Invitrogen) from which cDNA was generated using SuperScript II Reverse Transcriptase (Invitrogen). TaqMan qPCR was carried out for human Bax and GAPDH using TaqMan Gene Expression Assays (Bax: 4453320 and GAPDH: 4448892; Invitrogen) and ViiA 7 Real-Time PCR system (Life Technologies), respectively.

### Imaging GFP-tagged proteins

Parental CHO cells, or those stably expressing parkin (CHO-Parkin) or transiently transfected with WT parkin or R275W-Parkin were grown on coverslips and transfected with WT GFP-Bax, or the various GFP-tagged Bax mutants along with mito-mCherry to visualize mitochondria. Twenty-four hours after transfection, cells were treated with vehicle (DMSO) or staurosporine (1 *μ*M, 5 h). Cells were fixed using 4% paraformaldehyde, mounted onto slides and images were captured on a Zeiss Axio Imager M2 microscope (Carl Zeiss, Inc., Jena, Germany). Co-transfected cells were scored by visually categorizing them according to localization of Bax with respect to the mitochondrial marker mito-mCherry. They were categorized according to the degree of co-localization with mito-mCherry as ‘completely cytosolic', ‘predominantly cytosolic', ‘predominantly mitochondrial' and ‘completely mitochondrial', and plotted as a ratio to total cells counted. The experiments were performed in duplicate and repeated in three biological replicates, with a minimum of 46 cells counted per field.

### Immunocytochemistry

CHO and CHO-Parkin cells were grown on coverslips and transiently transfected with empty vector plasmid, untagged WT Bax, the ØK Bax or the K21R/K64R mutant Bax. Twenty-four hours after transfection, cells were treated with vehicle (DMSO) or staurosporine (1 *μ*M, 6 h). Cells were fixed with 4% paraformaldehyde, permeabilized with 0.1% Triton-X and immunostained for human Bax 2D2 (sc-20067; Santa Cruz Biotechnology) and stained with DAPI to visualize nuclei. Cy3-conjugated secondary antibodies were purchased from Jackson ImmunoResearch (West Grove, PA, USA). Coverslips were inverted onto slides and images were acquired using a Zeiss Axio Imager M2 microscope. Images were scored to quantify the different stages of Bax translocation into the mitochondria by a blinded observer as described above. All experiments were performed in triplicate and representative images are shown.

### Statistical analysis

Quantitative translocation of Bax was compared by one-way ANOVA followed by Tukey's multiple comparison test. A *P*-value of<0.05 was considered significant.

## Abbreviations

PD: Parkinson's Disease
CCCP: carbonyl cyanide 3-chlorophenylhydrazone
WT: wildtype
KO: knockout
PARP: poly ADP ribose polymerase
HEK: human embryonic kidney 293
CHO: Chinese hamster ovary
TOM20: translocase of outer membrane 20
VDAC: voltage-dependent anion channel
SDHA: succinate dehydrogenase complex-subunit A
RING: really interesting new gene
